# Methods of Primary Clinical Prevention of Dental Caries in the Adult Patient: An Integrative Review

**DOI:** 10.3390/healthcare11111635

**Published:** 2023-06-02

**Authors:** Nélio Veiga, Ricardo Figueiredo, Patrícia Correia, Pedro Lopes, Patrícia Couto, Gustavo Vicentis Oliveira Fernandes

**Affiliations:** 1 Faculty of Dental Medicine, Universidade Católica Portuguesa, 3504-505 Viseu, Portugal; 2 Centre for Interdisciplinary Research in Health (CIIS), Universidade Católica Portuguesa, 3504-505 Viseu, Portugal; gustfern@umich.edu; 3 Periodontics and Oral Medicine Department, University of Michigan School of Dentistry, Ann Arbor, MI 48109, USA

**Keywords:** primary prevention, adult, oral health, dental caries

## Abstract

Aim: Preventive approaches to oral health diseases, mainly dental caries, require individual and collective policies. Thus, this review was conducted to identify the primary prevention methods of dental caries in adults to improve oral health at the clinical and community levels. Methods: This review followed the PICO strategy with the research question: “What are the methods of primary prevention of dental caries, in adults, for improving and maintaining oral health integrating clinical and community-based strategies?” Electronic screening was carried out by two independent reviewers in five databases (MedLine/PubMed, SciELO, Web of Science, Cochrane Library, and LILACS) to find relevant publications between 2015–2022. We applied eligibility criteria for selection of the articles. The following MeSH terms were used: “Primary Prevention”; “Adult”; “Oral Health”; “Dental Caries”; “Fluorides, Topical”; “Fluoride Varnishes”; “Pit and Fissure Sealants”; “Preventive Dentistry”. Although the term “Prevention strategy” is not a MeSH descriptor, several correlated terms appeared and were used in the search engines: “Preventative Care”, “Disease Prevention, Primary”, and “Prevention, Primary”. The tool provided by the JBI organization (Joanna Briggs Institute) was used to assess the quality of the included studies. Results: Nine studies were included. Overall, it was found that the main primary prevention methods applied in dentistry in adults are the application of pit and fissure sealants, topical application of fluoride performed in the dental clinic, use of fluoridated toothpaste, mouthwash with chlorhexidine at home, use of xylitol, the recommendation for regular appointments with the dentist, and the need to inform patients about the saliva buffer capacity and adoption of a non-cariogenic diet. For that purpose, preventive policies should be taken to prevent dental caries. These include three major challenges: providing the adult population with more knowledge regarding their oral health, empowering patients through adopting healthy lifestyles, and developing new preventive strategies and awareness campaigns aimed at the adult population to promote proper oral health habits. Conclusions: A small number of studies were found whose participants were adult patients. There was some consistency regarding primary prevention methods in our studies. However, good quality randomized control studies are still required to define the best intervention strategies for adult caries prevention.

## 1. Introduction

### 1.1. Oral Health

The International Dental Federation (IDF) defines oral health as “multi-faceted and included the ability to speak, smile, smell, taste, touch, chew, swallow and convey a range of emotions through facial expressions with confidence and without pain, discomfort, and craniofacial complex diseases”. The IDF definition integrates oral health with general health, demonstrating that it affects overall health and well-being. Therefore, increasing awareness about the different dimensions of oral health and how they change over time empowers people by recognizing that values, perceptions, and expectations influence its outcomes [1]. The concept of oral health was extended with the addition of the feeling of well-being after the World Health Organization (WHO) expanded the definition of health by encompassing social well-being. Since then, oral health has been considered to also contribute to general well-being, not just the absence of pathologies. Everyday activities such as eating, talking, smiling, and contributing to society are included in a person’s well-being. Therefore, oral health is currently understood to be an integral part of overall health and well-being [2].

A paradigm shift has occurred regarding health, disease causation, and healthcare delivery in medicine and dentistry. The medical model has been replaced by the socio-environmental model of health, which assumes health status as the capacity for optimal functioning and social and psychological well-being. Thus, oral health is a comfortable and functional dentition allowing individuals to continue in their desired social role [3]. The primary responsibility for maintaining oral health lies with the individual or their caretakers, which must be achieved through effective, evidence-based self-care. Still, public health policy support, education, professional monitoring, and therapeutic interventions are necessary. Self-care becomes more effective when individuals have oral health literacy and thus demand a functional and aesthetically appealing dentition. Dentistry, therefore, has a vital role in promoting strategies and methods of primary prevention of dental caries for improving and maintaining oral health [4].

### 1.2. Primary Health Care and Its Promotion

The concept of primary health care was defined at the Alma-Ata conference in 1978. Primary care for oral health is an integral part of primary health care. In 1984, the European WHO Discussion Document [5] defined health promotion as follows: health promotion involves the population as a whole, in the context of their daily lives, as opposed to focusing on people at risk of specific diseases; it is directed towards action on the determinants or causes of health; it combines diverse but complementary methods or approaches; it aims at particularly effective and concrete public participation; and health professionals have an important role in sustaining and enabling health promotion [5,6].

Health promotion programs are effective if based on a holistic perspective of health, empowering citizens to manage their health, promoting equitable access to information, and leading to the adoption of healthy lifestyles [7]. Thus, several primary prevention methods applicable to oral health can be developed among adults, including behavioral factors, such as regular tooth brushing, dental flossing, a balanced diet, and regular dental checkups. These factors significantly reduce the risk of oral disease [8,9,10]. Currently, there is a paradigm shift in the treatment of dental caries, advocating a preventive approach, resulting in the conservation of dental structure [11,12].

There are two primary clinical preventive strategies for reducing the risk of dental caries: topical fluoride application and fissure sealant application. Topical fluoride application is a more effective method on smooth tooth surfaces, while pit and fissure sealants are used successfully on the occlusal tooth surface [13,14,15,16]. Pit and fissure sealants prevent oral bacteria and carbohydrates, from the diet, from accumulating in the cavities and fissures and developing an acidic environment essential for developing the dental caries process. One advantage of pit and fissure sealant application is related to the fact that this is an easy technique without the need for local anesthesia [9]. Due to their liquid and fluid consistency, sealants flow over the irregular surface of grooves and fissures, filling all the porosities present, sealing the regions that retain bacterial plaque and, as a result, preventing and/or delaying the appearance of occlusal dental caries. On the other hand, pit and fissure sealants are mainly characterized by their fluoride-releasing action [17,18,19,20].

Another approach to prevent caries is the use of xylitol. It has been used since the early 1960s in the diet of diabetic patients and, most recently, as a sweetener in products aimed at improved oral health [21,22]. Xylitol disrupts the energy production processes of mutans streptococci, leading to a futile energy consumption cycle and cell death. Moreover, it reduces plaque formation and bacterial adherence and inhibits enamel demineralization [23]. The first xylitol studies in humans showed the relationship between dental plaque and xylitol and the safety of xylitol for consumption [24]. The first chewing gum developed with the aim of reducing caries and improving oral health was released in Finland in 1975 [9]. Since then there have been various products introduced and sold over the counter and applied professionally worldwide. It has demonstrated to be an effective strategy as a self-applied caries preventive agent [21], and the recommended dose for dental caries prevention is 6–10 g/daily [25].

### 1.3. Fluoride Therapy

Fluoride has played an essential role in protecting enamel. Therefore, the relationship between fluoride and demineralization reduction is log-linear [26,27]. Evidence shows that fluoride is more effective than calcium [28] and can be found in various forms and concentrations, such as toothpaste, mouthwashes, gels, and varnishes [29]. Evidence reports that fluoride toothpaste (1000 to 1500 ppm) effectively reduces dental caries rates [30]. A systematic literature review [31] showed that fluoride toothpaste (1000 to 1250 ppm fluoride) was more effective than non-fluoride toothpaste in reducing dental caries rates. Fluoride mouthwash typically has 0.05% sodium fluoride, corresponding to a solution with approximately 230 ppm of fluoride [32]. In turn, fluoride varnishes have 5% sodium fluoride (22,600 ppm) as the active agent and can be used to prevent dental caries [33,34,35,36,37]. Fluoride varnish treatments effectively stop the progression of tooth demineralization, reducing dental caries significantly by approximately 50% to 70% in dental pits and fissures. Their effectiveness is even greater on interproximal surfaces. The beneficial effect of varnishes on permanent teeth is thus recognized [38,39,40]. Patients at high risk of dental caries, namely patients with xerostomia, and elderly patients at risk of root caries, may benefit from boosters to improve the remineralizing and preventive efficacy of fluoride [41,42].

### 1.4. Dental Caries

However, dental caries is the most prominent oral health problem affecting most adults, including in industrialized countries. The prevalence of dental caries in permanent dentition in Portugal has been decreasing significantly, as reported by national studies, reaching very satisfactory levels, particularly in individuals who benefit from the activities developed under the National Program for Oral Health Promotion (Programa Nacional de Promoção da Saúde Oral, PNPSO) [43,44].

The WHO’s global oral health assessment shows that while there has been a significant improvement in many countries, untreated dental caries is still a significant global burden: “The current pattern, within dental caries and periodontal disease, reflects the different risk profiles in different countries (the living conditions, lifestyles, and environmental factors) and, in particular, the result of the implementation of oral health promotion programs” [45,46]. One significant challenge today is the unmet treatment needs of adult patients, indicating limited access to dental care and insufficient primary prevention efforts. Therefore, the adult population still experiences significant complications due to untreated caries. Thus, more measures must be adopted by the NHS, as well as by health entities and professional organizations, to minimize the prevalence of dental caries in the world, particularly in Portugal [47].

Developing preventive interventions at the primary prevention level can lead to potentially sustainable normative support for oral hygiene, locally tailored and targeted approaches, and ongoing positive changes in specific oral health practices. Such interventions can reduce the short- and long-term psychosocial and economic costs associated with disabling oral health problems and help prevent the exacerbation of chronic and disabling diseases [48,49,50]. Nowadays, there are high-standard treatments for untreated caries [51], but this may negatively affect the NHS and patients either from a health or from an economic point of view [52].

Currently, primary prevention strategies should consider models of behavioral change and patient empowerment linking oral health to a healthy lifestyle, considering the following parameters: capacity, opportunity, motivation, the feeling of appreciation, behavior, and patient-centered strategies. Moreover, the current health perspective includes a holistic, global, and integrative approach in a predictive, preventive, personalized, and participatory vision. The biggest challenge for community oral health is to promote proactive strategies in adult patients. Therefore, collaboration work and stakeholder networking will be the key to success. This requires the effective use of education strategies [53,54,55,56,57].

Therefore, this review is an adjunct to understanding the main factors involved in patent neglect in this specific age group and may help define future strategies and recommendations to ensure greater efficacy and application of preventive treatments in adults. Then, the aim of this review was to gather the current available information on strategies and methods of primary prevention of dental caries in adults to improve and maintain oral health.

## 2. Materials and Methods

### 2.1. Search Strategy and Eligibility Criteria

The present review was registered in PROSPERO (CRD42021243161). According to the PI[C]O framework, the following research question was defined [58]: “What are the methods of primary prevention of dental caries (I) in adults (P) for improving and maintaining oral health integrating clinical and community-based strategies (O)?” The following eligibility criteria were defined and described in Table 1.

### 2.2. Study Search Strategy

For the identification of relevant studies in accordance with the defined criteria, searches will include studies published between 2015 and 2022 in Portuguese, Spanish, and English using the following electronic databases: MedLine/PubMed, SciELO, Web of Science, Cochrane Library, and LILACS. The exclusion of studies before 2015 is due to a similar article published in 2016 which included articles up to 2015 [16], and thus we are trying to provide results from more recent studies. The following MeSH terms were used, retrieved from MeSH Descriptor Data https://meshb.nlm.nih.gov/search (accessed on 22 January 2020): “Primary Prevention”—MeSH Heading; “Adult”—MeSH Heading; “Oral Health”—MeSH Heading; “Dental Caries” MeSH Heading; “Fluorides, Topical”—MeSH Heading; “Fluoride Varnishes” MeSH Heading; “Pit and Fissure Sealants” MeSH Heading; “Preventive Dentistry” MeSH Heading. Although the term “Prevention strategy” is not a MeSH descriptor, several correlated terms appeared and were used in the search engines: “Preventative Care”, “Disease Prevention, Primary”, “Prevention, Primary”. All these descriptors were conjugated with the Boolean operators “AND” and “OR”. In the mentioned scientific search engines, these descriptors were used in Portuguese, Spanish, and English for article retrieval.

### 2.3. Screening and Data Extraction

Two independent reviewers (NV and RF) were responsible for the searches and screening of the articles. After eliminating duplicates, the articles passed through 3 stages: (i) reading of the title, (ii) reading of the abstract, and (iii) reading the full text. In disagreement, a third reviewer (PC) broke the tie. The same independent reviewers selected data from each included article and recorded them in an Excel^®^ sheet (version 15.17, Microsoft, Redmond, Washington, DC, USA). Any potential disagreement and/or discrepancy was resolved by consensus and in the presence of a third reviewer (PC). The following variables were defined in this investigation: Authors and year of publication, title, study design, participants, objective, and results.

### 2.4. Quality Assessment/Risk of Bias

The tool provided by the JBI organization (Joanna Briggs Institute, University of Adelaide, North Adelaide, Australia) was used to assess the quality of the included studies. The purpose of this tool was to evaluate the methodological quality of research and determine the extent to which the possibility of risk of bias has been addressed in its design, execution, and analysis. It included 11 questions (final score ranging from 0 to 11, 0% to 100%), with the following responses: yes (1), no (0), undefined (UND), and not applicable (N/A). A value below 50% indicates a low quality of the article; between 50 and 69% corresponds to moderate quality, and ≥70% reflects a high quality [59].

## 3. Results

**Figure 1:**
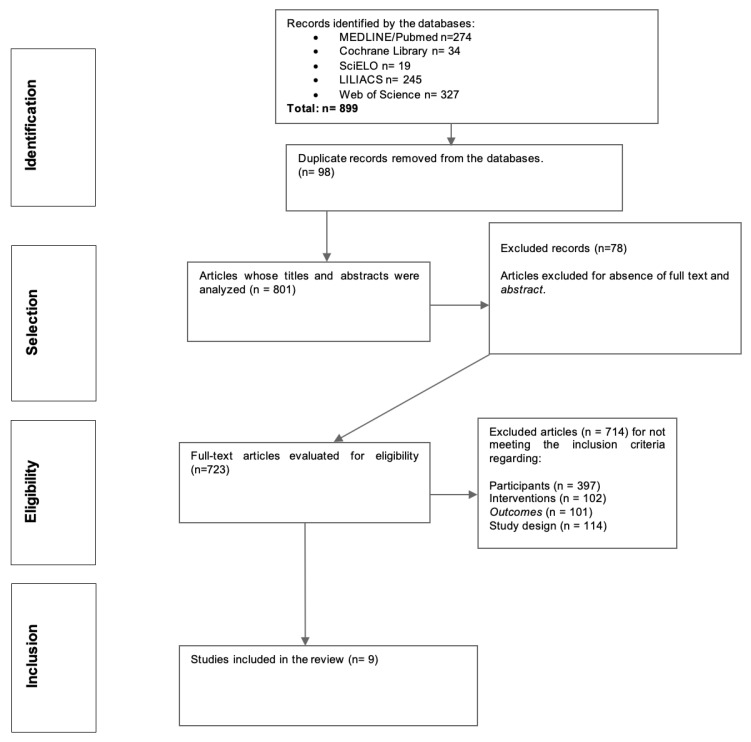
PRISMA flow chart.

A total of 899 articles were identified. In the first step, duplicate studies were removed from the databases (*n* = 98). In the second phase, out of 801 articles, 78 were excluded by reading their titles and abstracts, resulting in 723 articles. This large number was found because the title and the abstract did not provide enough information in order to exclude the article, which led us to include a great number for full-text reading. From these, 714 were excluded for failing to meet the other inclusion criteria, namely: 397 for participants (most studies conducted in children and adolescents; 102 for interventions; 101 because the results did not answer the research question; 114 whose study design did not fall within those recommended for a review of the literature, being mostly secondary studies). Thus, nine articles [60,61,62,63,64,65,66,67,68] were included in the study after applying quality assessment tools (k = 0.99) (Figure 1).

Table 2 summarizes the results of the nine studies included in this review, considering: authors/year of publication, article name, study design participants, objective, and results. The quality assessment showed (Table 3) that the articles included in this review had values greater than 70% and were considered high quality, except for one which presented results with lack of information and was of moderate quality.

## 4. Discussion

The present review answered the research question on the primary prevention of dental caries in adults. It permitted us to understand the improvement and maintenance of oral health at clinical and community levels, involving: (i) fluoride application in the office; (ii) recommending the use of fluoride toothpaste; (iii) performing mouth rinses with chlorhexidine at home; (iv) recommending the use of xylitol; (v) treatments with fluoride varnishes in dental pits and fissures; (vi) more frequent visits to the dentist; (vii) adopting a non-cariogenic diet, i.e., alerting patients to the frequency of sugar consumption; (viii) brushing correctly; and (ix) alerting patients to the protective role of saliva in dental caries.

The studies included in this review were mostly cross-sectional studies, with one retrospective cohort study and one qualitative study, presenting various strategies for primary prevention of dental caries in the adult population from different countries. The individual analysis showed significant homogeneity in the design of the studies, specially concerning cross-sectional studies. There was substantial heterogeneity regarding the sample, the variables studied, and the type of intervention applied, which made it impossible to perform a meta-analysis. Briefly, these studies included dental practitioners, dental students, dental committee members, and insurance company members, aiming to evaluate the preventive measures for dental caries in adult patients. Most studies considered the participants’ knowledge and awareness of preventive measures.

### 4.1. Types of Caries Prevention

It is known that there exist many types of caries prevention, such as dietary control, oral hygiene, topical antimicrobials, pit and fissure sealants, and fluoride therapy and supplements. A dietary diary can be considered in cases of moderate/high risk of caries, leading to a specific dietary counselling. Therefore, its compliance should be monitored in recall visits [68]. In addition, oral hygiene with tooth brushing is an efficient mechanical method to remove dental plaque. Skills of toothbrushing must be reviewed/taught and the patient should be encouraged to brush their teeth at least twice a day, especially before bedtime. Furthermore, the use of fluoridated toothpaste should be emphasized [69], which can reduce the risk of development of caries [68].

Fluoride mouthrinses can be also considered; however, this is not recommended in fluoridated communities and for children under 6 years old (risk of ingestion). The two main concentrations available are 0.05% (225 ppm F) NaF, and 0.2% (900 ppm F) NaF, for daily and weekly uses, respectively [68]. Fluoride gel is produced by the addition of a gelling agent, increasing its viscosity. It may lead to a 28% reduction in the risk of dental caries. The most used gel has 12,300 ppm of Acidulated Phosphate Fluoride (APF), which is applied to teeth by the tray technique [68]. Fluoride supplements are provided in the form of tablets, lozenges, drops, liquids, and fluoride-vitamin preparations. All potential fluoride sources should be evaluated, and a caries risk assessment should be conducted before prescribing them, to reduce the risk. At low caries risk, the supplements are not recommended, and other sources of fluoride should be considered as a preventive measure [70]. Fluoride therapy can have systemic and topical effects. In case of the water fluoridation, there is a release of F compound into a public water supply to bring the F ion concentration up to a level that effectively prevents caries. The optimal F concentration in drinking water is a range of 0.7–1.2 ppm, depending on the climate, according to the WHO guidelines. This method reduces dental caries experience by the half [71]. Those patients with higher risk of developing caries should use a standard of 1000 ppm paste [72].

Fluoride varnish is a professionally applied adherent material and is not intended to be as permanent as pit and fissure sealant. It is effective in preventing new carious lesions and halting the progression of established ones. The application of high F concentrations, around 22,000 mg F/L, leads to slow release of F into the surrounding environment. This release has been shown to continue for 5–6 months [70]. It typically contains 5% sodium F (NaF), which is equivalent to 2.26% F (Duraphat), and an organic F varnish which contains 0.1% F (Fluor Protector) is available. This is applied to teeth by the paint-on technique [73].

Sealant is a low viscous material that is placed in the pits and fissures on occlusal, buccal, and lingual surfaces of teeth to prevent or arrest the development of caries. Sealants have been used for over 30 years as a caries preventive measure and evidence from clinical trials has demonstrated their effectiveness [74]. Applying a fissure sealant decision should be made on clinical bases, after a full clinical examination that is supported by a caries risk assessment. Applying a sealant over an incipient carious lesion (noncavitated carious lesion) does not lead to progress if this sealant remains intact. However, if caries is found to extend to dentine, a restoration should be placed [73]. It is recommended to use the sealants compared with both nonuse of sealants and use of fluoride varnishes in permanent molars with both sound occlusal surfaces and noncavitated occlusal carious lesions. Nevertheless, sealant is contraindicated if patient behavior does not permit isolation, there is an open occlusal carious lesion, caries exists on other surfaces of the same tooth, a large occlusal restoration is already present, or if pits and fissures are well coalesced and self-cleansing [75].

The use of topical antimicrobials (chlorhexidine and xylitol) can help with prevention, reducing the burden of bacteria [76]. Chlorhexidine is the gold standard antibacterial agent which reduces the mutans streptococci levels. Several over the counter and professionally administered chlorhexidine-based preparations are available, as toothpastes, mouthrinses, varnishes, gels, and gums and sprays [77]. The use of xylitol can also reduce levels of caries-forming mutans streptococci in plaque and saliva. It can be administered in gum, lozenges, or snack foods [76].

### 4.2. Findings and Contrast with the Literature

Tagliaferro et al.’s study [60] investigated the procedures used by dentists in a Brazilian community to prevent the carious process in adult patients. They indicated that the procedures used were pit and fissure sealants, fluoride application in the office, the recommendation to use fluoride toothpaste, mouth rinses with chlorhexidine at home, and advice to use xylitol as a replacement for sucrose in the diet. This result agrees with Nassar’s study [61], which evaluated the awareness and knowledge of dental students regarding the preventive measures for dental caries in adult patients. As result, the students mentioned that the practical measures for the prevention of dental caries in patients include the recommendation of oral hygiene, xylitol, application of fluoride in the office and its use at home, use of chlorohexidine, and recommendations about the importance of a protective diet against dental caries. These findings coincide with Wagle et al.’s study [67]. The authors aimed to describe the practices of Nepalese dentists regarding preventive education and treatment in patients, where the dentists advised patients about the use of xylitol.

Similarly, Marchesan et al.’s study [62] assessed the associations between oral hygiene behaviors and the prevalence of dental caries, periodontal disease, and the number of lost teeth. In addition, they supported previous studies in that adult patients are recommended to use fluoride toothpaste and mouth rinses with chlorhexidine. In this study, associations between oral hygiene with variables (age, race, gender, diabetes, smoking, education, visits to the dentist, and regular sugar consumption) were found, leading to the proposal of other primary prevention measures to reduce these oral health risk factors. It recommended patients to visit the dentist more frequently and to adopt a non-cariogenic diet. The same study stated that patients with better oral hygiene and more visits had fewer cavities and teeth loss compared to those who lack oral health habits and have fewer visits to their dentist. Moreover, the study suggested that patients with daily oral hygiene habits had lower levels of periodontal disease and dental caries, as well as fewer lost teeth. It also found that a lower frequency of oral hygiene habits correlated with increased periodontal disease. Similarly, in Wagle et al.’s study [67], one of the measures to prevent dental caries was to alert patients to the frequency of sugar consumption.

Meless et al. [63] conducted a study on 400 patients with a mean age of 35.5 years (± 13.1 years). The most evident dental caries preventive measure were sealants and topical fluoride application. In addition, the study demonstrated a pressing need for the presentation, planning, and implementation of primary prevention measures for oral health in the dental office and a greater awareness in the population that they should not visit their dentist only when dental pain is present. By analyzing this study [63], it was possible to infer that preventive dentistry is not the patient’s primary goal when considering a dental appointment. Accordingly, the main reasons for a dental appointment were pain (91.5%) and esthetics (23.5%). The study states that oral hygiene was insufficient in 36.8% of the patients, and there was a high prevalence of dental caries disease (98.7%).

Leggett et al.’ study [64] aimed to qualitatively explore the perceived barriers and promoting factors of oral disease prevention, with a sample of adult patients who were members of the medical–dental team, members of dental committees and members of insurance companies in the UK, Denmark, Germany, the Netherlands, Ireland, and Hungary. The study showed that one of the main barriers to oral health promotion was the populations’ lack of financial capacity. This was a less problematic fact in Denmark, the Netherlands, and Germany, countries where prevention is reimbursed as provided care. Nevertheless, dentists believe this reimbursement is insufficient to implement more effective primary prevention measures. They suggested a major focus on education of the population about prevention and changing oral health behaviors, with a fairer and more equitable distribution of dental checkups. In all six countries, the need for change emerged, which implies effective joint work for more effective oral health promotion in those communities.

Applying sealants was also one of the preventive measures presented by Aledhari et al. [65], as well as warning patients about the “frequency versus amount of sugar consumption”. In this study, which enrolled 59 dentists working in Baghdad, there was the assessment of the preventive orientation of Iraqi doctors, in terms of “knowledge” and “attitude” towards dental caries prevention and regarding their “preventive practice”. The results showed a significant association recorded between continuing professional education and preventive advice on dental caries in the adult population (*p* = 0.03).

Arheiam and Bernabé [66] evaluated the attitudes and practices related to dental prevention among Libyan dentists, in addition to the recommendation of good oral hygiene and the use of fluoride toothpaste. It was also found that the preventive measures performed by dentists were the application of sealants, a preventive measure for the carious process that was transversal to the previous studies, as well as treatments with fluoride varnishes in dental pits and fissures. Sealant placement is also present in Wagle et al.’s study [67].

Nishi et al. [78] aimed at appraisal of the knowledge regarding dental caries risk factors/indicators in two groups of adult patients with different socioeconomic profiles, from two culturally distinct countries (Japan and Ireland). In this study, in addition to the Irish dentists finding that patients did not periodically attend checkups and use sodium fluoride, the authors compared them to the patients of the Japanese dentists. Both groups indicated that patients did not brush their teeth properly and were unaware of the protective role of saliva on tooth decay. Moreover, the research also revealed a transversal lack of knowledge of patients regarding periodic checkups and the use of fluoride to prevent dental caries.

In order to improve and maintain oral health, our results corroborated the methods of primary prevention of dental caries in adults, which can be seen in evidence found in the literature. This stated that the main strategy consisted of topical application of fluoride and sealants in pit and fissure [13,14,15,20,33]. The beneficial effect of varnishes on permanent teeth was also recognized [38], which was confirmed by Wagle et al. [67]. Petersson et al.’s [57] and Damle’s [79] studies supported that primary prevention includes topical fluorides application (fluoride gel) [80,81], fluoride varnish [80,81], fluoride oral paste [82], tooth brushing, and nutritional counselling. All decreased/excluded potential dental caries cavities and risk. The same authors also supported the promotion of dental prophylaxis, based on the encouragement/education of a correct oral hygiene and regular visits to the dentist, not only in the presence of pain, which was observed in the present review.

One fact was observed in the scientific literature, which was lack of consensus about gender (of the professional staff) differences about attitudes on prevention and treatment of dental caries. Some articles pointed out that a more conservative approach was found among female dentists [83,84,85,86]. On the other hand, other studies found no statistically significant relationship between dentist gender and choices for caries prevention or treatment [87,88,89]. Further studies are necessary to clarify this issue. Other factors for recommending some type of preventive method showed that patients ≥65 years old were more likely to receive in-office fluoride. This fact depends on the dentist and their concerns about root caries prevention, which affects four out of ten adults [90]. Pentapati et al. (2019) [90] showed an estimated prevalence of 41.5% for root caries; the authors suggested the application of preventive measures focusing on policymakers and healthcare professionals could reduce the possible future burden.

Furthermore, even though there is lack of literature supporting the cost-effectiveness for the use of fluorides and sealants on prevention in adult patients [91], it is possible to speculate that the patient’s interest in prevention can be more attractive than patient’s caries risk. Furthermore, Tagliaferro et al. [60] showed that only 34% of dentists were worried with the caries risk assessment. Another explanation may be that dentists are working in a person-centered care environment, employing the shared decision-making principles [92], in which the patient acts as a partner in the care delivery [93]. Further studies are necessary to evaluate these assumptions.

### 4.3. Limitations of the Study

As limitations, it is possible to refer to the limited number of articles focused on dental caries prevention in the adult patient, limited number of participants related to an extremely simple and significant disease studied, and possible correlation of this disease with other systemic problems. Furthermore, our study is an integrative review, which used a methodology to provide synthesis of knowledge and applicability of results of significant studies to practice; nevertheless, it had some limitations regarding the inclusion of studies with different variables, harming, sometimes, the comparison between studies’ data.

## 5. Conclusions

Within the limitations of this review, it was possible to conclude that the main factors and difficulties associated with the application of primary prevention strategies for dental caries in adults were not related to the dentist’s lack of knowledge, but to inadequate attention paid to the importance of preventive dentistry with their patients. This absence of primary care combined with the adult population’s lack of knowledge about this topic resulted in a higher rate of invasive treatments (dental restorations, endodontic treatments, or dental extractions). Based on the results of this review, more studies should be developed in order to check the application of primary prevention methods in other countries and populations, to provide the best information for the oral health.

## Figures and Tables

**Table 1:** Inclusion and exclusion criteria for study selection.

Selection Criteria	Inclusion Criteria	Exclusion Criteria
[P] Participants	People aged ≥18 years	Underage individuals
[I] Intervention	Primary prevention strategies for dental caries	Studies that analyze other variables
[C] Comparisons/context study	Not applicable	Not applicable
[O] Outcomes	Improving and maintaining oral health at clinical and community levels	Studies that only analyze the effect of secondary and tertiary prevention measures
*Study design*	Cross-sectional studies, exploratory cross-sectional studies, prospective cohort studies, descriptive correlational studies, randomized controlled studies, cross-sectional comparative studies, cohort studies, qualitative studies	Reviews, systematic reviews and meta-analysis, comments, expert opinion, in vivo (pre-clinical) study, in vitro study
Publication date	2015–2022	Before 2015
Languages	Portuguese, Spanish, and English	Other languages
Study design	Clinical study; randomized clinical trial	

**Table 2:** Summary of the included studies.

Authors	Title	Study Design	Participants	Objective	Findings
Study 1 (S1) Tagliaferro, Silva, Rosell, Junior, Riley, Gilbert, and Gordan [60]	Methods for caries prevention in adults among dentists from a Brazilian community	Cross-sectional descriptive study	Dental physicians (*n* = 197) reported that at least 10% of their patients were >18	To investigate the procedures used to prevent dental caries in adult patients by dental physicians in a Brazilian community	Preventive measures for dental caries in adult patients: pit and fissure sealants; fluoride application in the office, recommended use of fluoride toothpaste; chlorhexidine rinses at home; recommended use of xylitol (chewing gum, gum, toothpaste + sodium fluoride, toothpaste + sodium fluophosphate, candies, as a replacement for sucrose in the diet)
Study 2 (S2) Nassar, H.M. [61]	Dental Caries Preventive Considerations: Awareness of Undergraduate Dental Students	Cross-sectional study	118 dental students from Turkey	To assess dental students’ awareness and knowledge of preventive measures for dental caries in adult patients	The students indicated practical measures to prevent dental caries in patients: oral hygiene, xylitol, fluoride application in the office and its use at home, use of chlorhexidine, and dietary factors. However, 40% of the students reported formative needs and training regarding diagnosis, preventive agents of dental caries, and risk-based treatment plans. Awareness of the need for more training in risk-oriented prevention of dental caries appears to predict increased self-perceived skills and knowledge of students
Study 3 (S3) Marchesan, J.T., Morelli, T., Moss, K., Preisser, J.S., Zandona, A.F., Offenbacher, S., Beck, J. [62]	Interdental Cleaning Is Associated with Decreased Oral Disease Prevalence	Retrospective cohort study	Data from 6891 adult patients (≥30 years) are available from the National Health and Nutrition Examination Survey (NHANES; 2011 to 2012 and 2013 to 2014)	To evaluate the associations between oral hygiene behaviors and the prevalence of dental caries, periodontal disease, and the number of lost teeth	Patients with better oral hygiene and more visits to their dental doctor had fewer coronal caries, interproximal coronal caries, and lost teeth compared to those with fewer oral health habits and fewer visits to their dental doctor (*p* < 0.0001). The latter were 1.73-times (95% confidence interval, 1.53 to 1.94) more likely to have ≥ 1 coronary caries surface than those in the other group. Patients with daily oral hygiene habits, as recommended by their dentist, had lower levels of periodontal disease and dental caries, as well as fewer lost teeth. A lower frequency of oral hygiene habits correlated with increased periodontal disease. The data support fluoride toothpaste; chlorhexidine mouth rinses for oral health promotion. Considering the existence of associations between oral hygiene with age, race, gender, diabetes, smoking, education, visits to the dentist, and regular sugar consumption, it was also proposed as a primary prevention measure for the reduction of these oral health risk factors, as well as more frequent visits to the dentist and the indication of a non-cariogenic diet. These measures were assumed to be essential resources for a system with a preventive focus on oral diseases, namely dental caries
Study 4 (S4) Meless, G.D., Guinan, J.C., Sangaré, A.D., N’Guessan, K.S., Kouakou, K.L., Da-Danho, V., Datté, A.S., Nouaman, N.M., Amangoua, A., Samba, M., Bakayoko-Ly [63]	Oral epidemiological profile of patients attending public oral health services in Haut Sassandra region, in Côte d’Ivoire	Cross-sectional study	400 patients (51.5% male) were observed, with a mean age of 35.5 years± 13.1 years	To determine the type of care and the epidemiological profile of patients seen in the 3 public medical-dental offices in the Haut-Sassandra region of Ivory Coast	The main reasons for consultation were pain (91.5%) and aesthetics (23.5%). Oral hygiene was insufficient in 36.8% of the patients. The oral conditions were malocclusions (12.8%), dental caries (98.7%), and edentulism (65.7%), with only 11.8% of the patients having dentures. The average CPOD index (index of decayed, missing, and filled permanent teeth) was 9.3. Dental extractions and placement of fixed dentures were observed in all 3 healthcare facilities. Preventive dentistry consisted of sealants and topical application of fluoride. The most commonly performed medical acts were extractions (74.5%). The results of this study highlight the need to plan primary prevention measures for oral health in the dental office and to raise awareness among the population, who should not visit their dentist only when dental pain is present
Study 5 (S5) Leggett, H., Csikar, J., Vinall-Collier, K., Douglas, G.V.A. [64]	Whose Responsibility Is It Anyway? Exploring Barriers to Prevention of Oral Diseases across Europe	Qualitative study	58 interviews and 13 focus groups were conducted involving 149 participants from the UK, Denmark, Germany, the Netherlands, Ireland, and Hungary. Participants were patients (*n* = 50), members of the medical-dental team (*n* = 39), and members of dental committees (*n* = 33) and insurance companies (*n* = 27)	To qualitatively explore the perceived barriers and promoting factors of oral disease prevention from a multiple participation perspective in 6 European countries	Five themes emerged at the level of barriers and factors promoting oral health in the community: better medical-dental guidelines for increased and sustained prevention of oral diseases; patients’ knowledge and motivation to follow these guidelines; trust in dentists; sociodemographic factors. All participating countries addressed these themes; however, the differences between countries were evident in the magnitude of each theme. One of the main barriers to oral health promotion was the populations’ lack of financial capacity. However, this is less of a problem in countries such as Denmark, the Netherlands, and Germany, where prevention is reimbursed as provided care. However, dentists still feel this reimbursement is insufficient to implement more effective preventive measures. The focus with the greatest consensus was on educating the population regarding prevention and changing oral health behaviors, along with a fairer and more equitable distribution of dental vouchers, which can significantly contribute to the greater prevention of oral diseases. The results suggest that in the 6 countries, primary oral health prevention is hampered by a complex interplay of factors, with no particular oral health system offering greater patient care. The need for change has emerged, which involves more teamwork to promote oral health in communities
Study 6 (S6) Aledhari F, Sargeran K, Gholami M, Shamshiri AR [65]	Preventive Orientation of Iraqi Dentists in Baghdad in 2016	Cross-sectional study	59 dentists working in Baghdad during the summer of 2016	To evaluate the preventive orientation of Iraqi dentists in terms of “knowledge” and “attitude” towards dental caries prevention and explore their “preventive practice”	Of all the respondents, 71% were women. The mean age was 40.75 ± 9.88 years (range 27–65 years). The most positive attitude toward preventive dentistry was related to the question, “Preventive dentistry is essential to the community” (*n* = 75, 83%). Higher reported knowledge on three questions: “frequency vs. the amount of sugar consumption,” “effect of sealants in preventing dental caries,” and “effect of oral health problems on overall health” (*n* = 83, 92.2%). Regression analysis showed a significant association between attendance at continuing education on preventive practice (*p* = 0.03)
Study 7 (S7) Arheiam A, Bernabé, E. [66]	Attitudes and practices regarding preventive dentistry among Libyan dentists	Cross-sectional study	166 dental doctors practicing in Benghazi	To assess attitudes and practices related to preventive medical-dental care among Libyan dentists	The dentists mentioned that preventive dentistry is very useful and essential for preventing oral diseases in the community. As for the orientations given to patients for good oral health, the most referenced were oral hygiene recommendations to use fluoride toothpaste; the preventive measures performed by the dentists were the application of sealants and fluoride varnish treatments in dental pits and fissures
Study 8 (S8) Wagle, M., Acharya, G., Basnet, P., Trovik, T.A. [67]	Knowledge about preventive dentistry versus self-reported competence in providing preventive oral healthcare—a study among Nepalese dentists	Cross-sectional study	195 dentists (71 men and 124 women)	To describe the practices of Nepalese dentists regarding preventive education and treatment in their patients; to assess their level of knowledge about preventive oral health	More than 90% of dentists considered themselves competent in preventive treatment and oral hygiene education for their patients. Female dentists demonstrated more intervention in oral disease prevention and oral hygiene promotion than men (*p* = 0.045). More than 70% of the dentists had good knowledge regarding the use of fluoride as a measure to prevent dental caries, as well as good knowledge about other aspects of oral health, such as alerting patients to the frequency of sugar consumption, use of xylitol, frequent visits to the dentist, placement of sealants. The vast majority of the participating dentists revealed a high overall competence in providing preventive treatment and education for their patients regarding oral health
Study 9 (S9) Nishi, M., Harding, M., Kelleher, V., Whelton, H., Allen, F. [68]	Knowledge of caries risk factors/indicators among Japanese and Irish adult patients with different socioeconomic profiles: a cross-sectional study	Cross-sectional study	The Japanese study involved 482 patients (aged ≥20 years) of 52 dental practitioners in a national initiative based on the Promoting Scientific Assessment in Prevention of Tooth Decay and Gum Disease (PSAP); the Irish study involved 159 patients (aged 20–69 years) with state-provided access (‘medical record’) of medical-dental services from eight practices in County Cork	To evaluate the knowledge about dental caries risk factors/indicators in two groups of adult patients with different socioeconomic profiles from two culturally distinct countries (Japan and Ireland)	The higher percentage value of Irish dentists who identified that patients ‘Do not visit the dental doctor for periodic check-ups’ (OR 2.655; 99% CI 1.550, 4.547) and ‘Do not use sodium fluoride’’ (OR 1.714; 99% CI 1.049, 2.802), compared to Japanese dentists. Both studies reveal that patients ‘Do not brush their teeth properly’ is a risk factor for dental caries and that they are unaware of the buffering capacity of saliva as a protective factor for dental caries. The study reveals: a lack of knowledge in Japanese patients: in performing periodic check-ups and the use of fluoride for the prevention of dental caries; in Irish patients, lack of understanding of the buffering effect of saliva as a protective factor against dental caries. In both groups, the need to inform patients regarding the protective effect of saliva emerged

**Table 3:** JBI quality assessment.

Author et al.	1	2	3	4	5	6	7	8	9	10	11	Total	%
Tagliaferro et al. [60]	1	1	1	0	0	1	1	1	1	1	1	9	81.81
Nassar [61]	1	1	1	1	1	1	1	1	1	1	1	11	100
Marchesan et al. [62]	1	1	1	1	1	1	1	1	1	1	1	11	100
Meless et al. [63]	1	1	1	0	0	1	1	1	1	1	1	9	81.81
Leggett et al. [64]	0	1	1	1	0	1	1	0	1	1	0	7	63.63
Aledhari [65]	1	1	1	1	1	1	1	1	1	1	1	11	100
Arheiam & Bernabé [66]	1	1	1	1	1	1	1	1	1	1	1	11	100
Wagle et al. [67]	1	1	1	1	1	1	1	1	1	1	1	11	100
Nishi et al. [68]	1	1	1	1	1	1	1	1	1	1	1	11	100

Values up to 50% indicates a low quality of the article; between 50 and 69% corresponds to average quality, and ≥70% reflects a high quality. (yes [1], no [0], undefined [UND], and not applicable [N/A]).

## Data Availability

The data used to generate and support the findings of this study are available from the corresponding author upon request.
